# Deflection of Resilient Materials for Reduction of Floor Impact Sound

**DOI:** 10.1155/2014/612608

**Published:** 2014-10-28

**Authors:** Jung-Yoon Lee, Jong-Mun Kim

**Affiliations:** ^1^Department of Civil and Environmental System Engineering, Sungkyunkwan University, Suwon 440-746, Republic of Korea; ^2^Department of Global Construction Engineering, Sungkyunkwan University, Suwon 440-746, Republic of Korea

## Abstract

Recently, many residents living in apartment buildings in Korea have been bothered by noise coming from the houses above. In order to reduce noise pollution, communities are increasingly imposing bylaws, including the limitation of floor impact sound, minimum thickness of floors, and floor soundproofing solutions. This research effort focused specifically on the deflection of resilient materials in the floor sound insulation systems of apartment houses. The experimental program involved conducting twenty-seven material tests and ten sound insulation floating concrete floor specimens. Two main parameters were considered in the experimental investigation: the seven types of resilient materials and the location of the loading point. The structural behavior of sound insulation floor floating was predicted using the Winkler method. The experimental and analytical results indicated that the cracking strength of the floating concrete floor significantly increased with increasing the tangent modulus of resilient material. The deflection of the floating concrete floor loaded at the side of the specimen was much greater than that of the floating concrete floor loaded at the center of the specimen. The Winkler model considering the effect of modulus of resilient materials was able to accurately predict the cracking strength of the floating concrete floor.

## 1. Introduction

Residential high-rise buildings have been gaining increasing popularity in densely populated countries due to their favorable properties. High-rise apartment buildings have the advantage of effectively using a relatively small land area, whereas they are also often disadvantaged by their housing environment. One of the most significant residential environment concerns in living in apartments is noise pollution due to floor impact sounds caused by footsteps, falling objects, moving furniture, and so forth.

In reinforced concrete apartment buildings, the floor impact sound from the floor above can easily transfer to the apartment below. Due to interior dry walls and thin floors, neighboring occupants can often hear each other's conversations. In the case of Korea, which has a population density of 503 person/km^2^ in 2011, the third most densely populated country in the world, the number of environmental grievances due to floor impact sound occurring in apartments is rapidly increasing: 12% in 2008, 18% in 2009, 28% in 2010, and 35% in 2011 of the total number of environmental grievances. The communities have established several standards on floor soundproofing to reduce the impact noise of apartments. The Apartment Housing Performance Grade Indication System currently enforced in Korea classifies the sound performance of apartments into several grades according to floor impact sound. In accordance with the dynamic stiffness measurement method of Korea Standard [1] that is based on the Japanese Industrial Standard [2] and ISO 9052-1 [3], the maximum light-weight impact sound and heavy-weight impact sound allowable were enforced to be less than 58 dB and 50 dB, respectively, in Korea. In case of Japan, the maximum light-weight impact sound and heavy-weight impact sound are allowed up to 60 dB. On the other hand, Australia Environment Protection Act [4] allows the maximum light-weight impact sound from 50 dB to 43 dB.

Two types of floor soundproofing solutions are available to reduce impact noise: acoustic matting and floating floors. Both solutions can reduce impact noise transferring through wooden or concrete floor structures. In the case of floating concrete floors, resilient materials are normally placed between the concrete or wooden slab and finishing materials. The resilient materials may effectively reduce sound impact through a floor by reducing the vibration caused as an item hits the floor. The level of noise that will be transmitted through the floor depends on the force of the impact, the vibration transmission characteristics of the floor structure, and the floor covering. Elmallawany [5] investigated the sound insulation in building and indicated that the sound insulation between rooms depended partly on the direction of sound propagation (which depended on the properties and dimensions of the partition between rooms). Sound insulation between rooms also partly depended on the flanking paths which involve the properties of the two facades and their thicknesses as well as on the specification and relative area of windows. Buratti and Moretti [6] investigated the impact sound insulation performances of materials used as floor coverings in buildings to reduce impact noise. Test results indicated that floating concrete floors must be carefully installed to reduce the annoyance caused by impact sound transmission between rooms in residential buildings. Kim et al. [7] tested eighteen types of resilient materials subjected to 24-hour load and 2-hour load. The result showed that the dynamic stiffness of resilient materials increased rapidly up to a 2-hour load. Experimental and numerical tests for floating floor systems with resilient materials by Cho [8, 9] indicated that the impact sound level peaks occurred at low frequencies due to in situ floating floor matched resonances.

Many studies on the effects of the types and density of resilient materials, dynamic stiffness, size of windows, wall types and materials, and different numbers of stories have been performed to examine the sound insulation of buildings [10–12]. On the other hand, few test results are currently available regarding the deflection of floor sound insulation systems. It is generally accepted that floor impact sound reduction increases with the decrease of dynamic stiffness of resilient material. Kim et al. [13] tested 51 resilient materials in order to investigate the correlation between the dynamic stiffness of resilient materials and their heavy-weight impact sound reduction level. Test results indicated that dynamic stiffness, as a physical property of resilient materials, decreased as the thickness of the resilient materials increased. If resilient materials with low dynamic stiffness are layered on top of resilient materials with high dynamic stiffness, the dynamic stiffness of the layered structure is similar to that of the resilient materials with low dynamic stiffness. Test results [12, 13] in the literature indicated that the density and the slab contact area of materials increased the dynamic stiffness of the material. Therefore, soft resilient materials with low modulus of elasticity or resilient materials with small slab contact area are commonly used to reduce dynamic stiffness.

**Figure 1:**
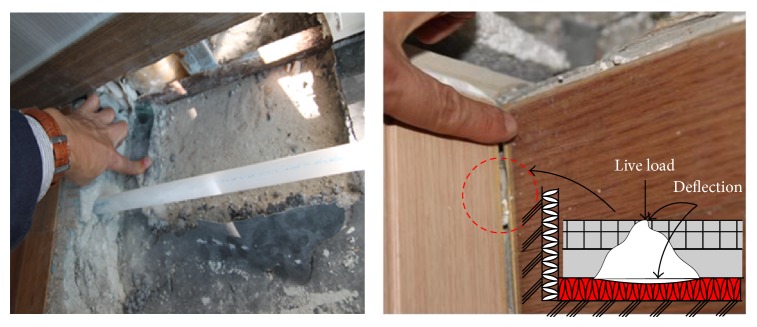
Deflection of floating floors in an apartment building.

The placing of resilient materials between the reinforced concrete slab and finishing mortar should not only reduce the floor impact sound vibration from the floor but also support the load on the floor. Thus, even if soft resilient materials satisfy the maximum limitation of light- and heavy-weight impact sound, these materials may not support the load on the floor. The density of material increases the dynamic stiffness of the material that is influenced by mass per area. The test results [12] indicated that the dynamic stiffness of the resilient material decreases its noise absorbing capacity. As a result, a stiff material with high density is less effective in absorbing floor sound than a soft material with low density. Therefore, soft resilient materials or resilient materials with a small slab contact area are commonly used to reduce dynamic stiffness. However, the soft resilient material also has to have some stiffness to a certain degree to support the load on the floor. If it is too soft or the contact with the bottom surface of the slab is too small, cracks may develop on the finishing mortar.  Figure 1 shows such a case, where the deflection of the floating concrete floors in an apartment building induced cracks on the finishing mortar and sinking of the finishing floor.

Since noise caused by movement in above apartments can adversity effect on the amenity of residents, the floor sound insulation systems are considered in design in residential apartments. Soft resilient materials in floor sound insulation systems may not support the load on the floor and cause to crack on the finishing mortar and sink the floor. To investigate this, experimental and analytical investigation on the deflection of floating floor sound insulation systems was conducted using a total of twenty-seven material specimens and ten floating concrete floor specimens in this study. In addition, as a classical method [14, 15] to calculate the deflection of floors normally having an intention of predicting the deflections of a stiff material, such as reinforced concrete and timber, an analytical method to calculate soil foundation was adopted in this study to calculate the deflection of floating floor systems. It is thus proposed that the current study, particularly findings on the cracking strength of floating floor systems, may provide useful information on the applicability of resilient materials on the floating floor systems.

## 2. Material Test

**Figure 2:**
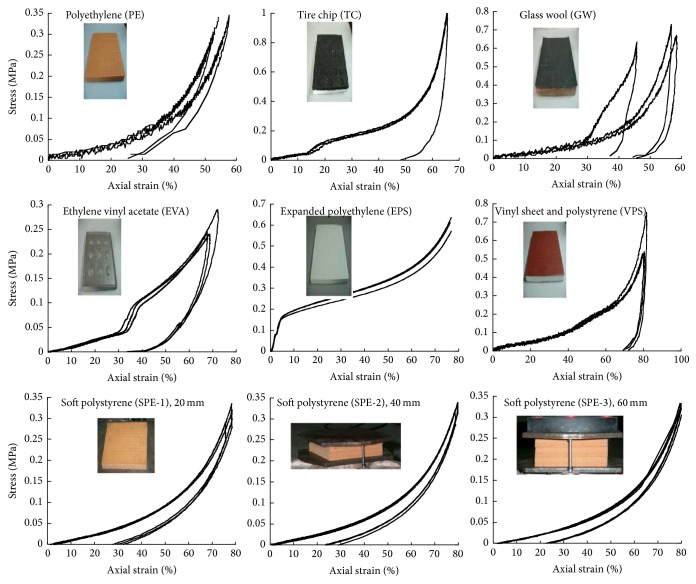
Stress versus strain curves of resilient materials.

A total of twenty-seven specimens were tested to measure the stress versus strain curves of resilient materials. Three parameters were considered in this investigation: types of materials, bottom shapes, and thickness. Seven types of materials for reducing floor impact sound were tested: polystyrene (PE), tire chip (TC), glass wool (GW), ethylene vinyl acetate (EVA), ethylene polystyrene (EPS), vinyl sheet and polystyrene (VPS), and soft polystyrene (SPE). Three nominally identical specimens were prepared for each specimen type as shown in Table 1. The materials are subdivided into two types of bottom shapes: plat shape and embossed shape (see Figure 2). The cross-sectional dimension of the specimens was a 100 mm square, and the thicknesses of the specimens were 20 mm, 40 mm, and 60 mm. The specimens SPE-1, SPE-2, and SPE-3 have the same material but different thicknesses. Deflection of the specimen was measured using four linear variable differential transducers (LVDTs) placed vertically at the bottom of the specimen at 90 degrees apart. The load was measured with the electronic load cell of the machine. The readings of the applied load and the corresponding LVDTs were recorded automatically through a data logger at specified load intervals. Figure 2 shows the compressive stress versus strain curves of tested specimens. Test results indicated that the strength of resilient materials was strongly influenced by the types of materials and the bottom shapes of materials. The stress versus strain curves of plat-shaped specimens differ from those of the embossed-shaped specimens. The compressive stress of plat-shaped specimens steadily increased with change in strain, while the curves of the embossed-shaped specimens are divided into two stages. At the beginning of loading, the compressive stress of the embossed-shape specimen increased with large change in slip because only the protruded part of the embossed sectional area resisted against the compressive load. After the cross-sectional area resists the compressive load, the stress of the embossed-shaped specimen rapidly increases with the increase of strain. The comparison of the curves, SPE-1, SPE-2, and SPE-3, having the same material but different thicknesses, indicates that the thickness of specimens does not affect the stress versus stress curve of the material. Table 1 shows the tangent modulus of curves at the strains of 0.1., 0.2, 0.3, and 0.4 of the tested specimens. As shown in Table 1, the tangent modulus of all specimens except EPS is approximately constant up to the strain of 0.3. Therefore, in this study, the secant line from zero stress to the stress corresponding to the strain of 0.3 was considered the tangent modulus of the material. In addition, the average value of the modulus at the strains of 0.1, 0.2, 0.3, and 0.4 was used for the tangent modulus of EPS. Test results [12] also indicated that the finishing mortar and the aerated concrete of the floor sound insulation system are generally cracked before the strain of material arrived at 0.3.

Aerated concrete laid between the resilient material and finishing mortar was prepared in accordance with the KS F 4039 standard [16]. The compressive strength and modulus of elasticity of aerated concrete were approximately 3.5 MPa and 220 MPa, respectively. Finishing mortar placed on the aerated concrete was prepared. The compressive strength and modulus of elasticity of finishing mortar were approximately 20.0 MPa and 1,600 MPa, respectively.

## 3. Floating Concrete Floor Test

### 3.1. Test Program

**Figure 3:**
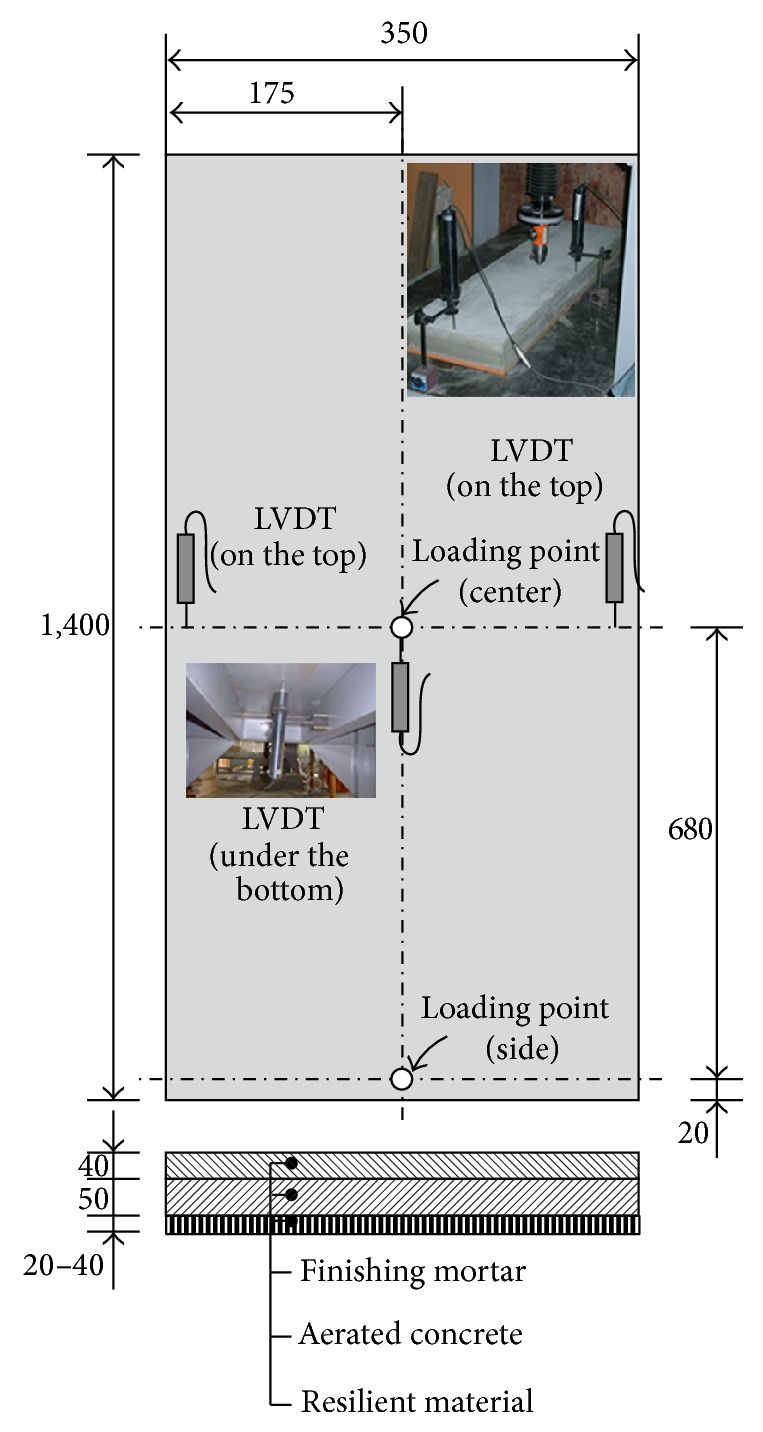
Dimensions and measurement of the floating floor specimens.

Floating concrete floors for RC apartment buildings generally consist of three materials: resilient materials, aerated concrete, and finishing mortar, as shown in Figure 3. The thicknesses of resilient materials, aerated concrete, and finishing mortar are 20~40 mm, 50 mm, and 40 mm, respectively. The specimens were 350 mm wide and 1,400 mm long. Aerated concrete and finishing mortar were cast on the resilient material in the horizontal position inside the timber formwork. After molding, the specimens were initially cured by covering them with fabric sheet, which prevented moisture loss for 24 hours. Immediately after the removal of the molds, specimens were cured in accordance with ASTM Standard C 511 [17] until the time of the test. During this curing period, they were sprayed with water two times a day to maintain moisture on the surfaces at all times. Two parameters were considered in this investigation: the types of resilient materials (seven types of material: polystyrene (PE), tire chip (TC), glass wool (GW), ethylene vinyl acetate (EVA), ethylene polystyrene (EPS), vinyl sheet and polystyrene (VPS), and soft polystyrene (SPE)) and the location of loading (center or side of the specimen). The material properties and dimensions of specimens F-PE2-1 and F-PE2-2 as well as F-SPE-1 and F-SPE-2 are the same. However, the concentrated load was applied at the center of specimens F-PE2-1 and F-SPE-1, while it was applied at the side of specimens F-PE2-2 and F-SPE-2. The properties of the specimens are shown in detail in Table 2.

### 3.2. Test Results

**Figure 4:**
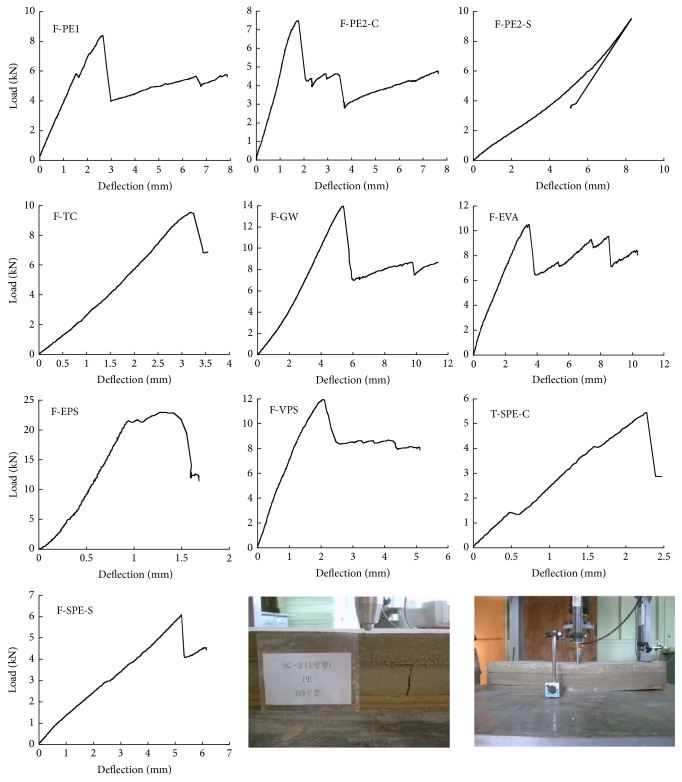
Load and deflection curves of the tested floating floors.

The structural behavior of ten floating concrete floor specimens was observed from the experimental tests. All of the specimens failed due to the cracking of the finishing mortar and the splitting of the aerated concrete. None of the specimens showed the local failure at the residual material. The stress of the tested specimens showed gradual increments with the increase of deflection, as shown in Figure 4. With increasing load, the slope of the load versus deflection curve was almost constant until the load reached the maximum. After the peak load, deflection significantly increased with decreasing load. The rate of load reduction of the PE specimens with respect to increasing deflection was greater than that of the EPS specimen but was similar to that of the TC and GW specimens. A greater decrement in slope was observed with the specimens loaded at the side (specimens F-PE2-S and F-SPE-S) than with that at the center (specimens F-PE2-C and F-SPE-C), yielding a higher deflection of specimens F-PE2-S and F-SPE-S at the maximum load than that of specimens F-PE2-C and F-SPE-C. The deflections corresponding to the maximum load of F-PE2-C and F-SPE-C were 8.22 mm and 5.21 mm, respectively, while those of F-PE2-C and F-SPE-C were 2.30 mm and 2.27 mm, respectively.

**Figure 5:**
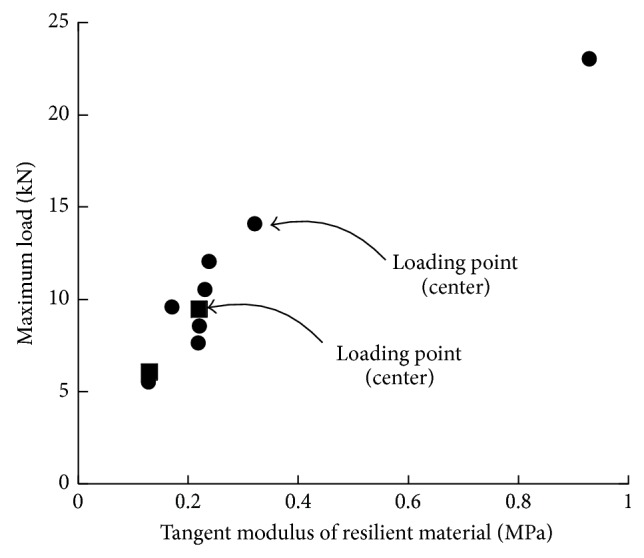
Relationship between maximum load of floating floors and tangent modulus of resilient materials.

The maximum load significantly increased with increasing the tangent modulus of the resilient material, as shown in Figure 5. The maximum load is positively proportional to the tangent modulus of the resilient material for all ten specimen types; the degree of proportionality was the highest, with F-EPS following in the order of F-GW, F-VPS, F-EVA, F-TC, F-PE, and F-SPE. The bottom shapes (plat shape and embossed shape) rarely affected the maximum load of the test specimen. The deflection of the floor loaded at the side is greater than that of the floor loaded at the center, and this can be attributed to the decreasing likelihood of the supporting area against the external load. A complete list of the maximum load and the deflection corresponding to the maximum load obtained from the test specimens is given in Table 2.

## 4. Prediction of the Deflection of Floor Sound Insulation Systems

In order to perform the numerical analysis for predicting the deflection of sound insulation systems, the Winkler model [18] was used in this study. This analytical model is normally used to investigate the deflection of members supported by a continuous constant stiffness foundation. Because the Winkler model is relatively accurate and simpler method than a FE analysis to predict the deflection of sound insulation systems, this model was adopted in this study. The modulus of elasticity of the reinforced concrete slab and the finishing mortar is much greater than that of the resilient material that is placed between the reinforced concrete slab and the finishing mortar. Therefore, it can be assumed that the resilient material supports the finishing mortar as a continuous constant stiffness foundation in the sound insulation systems.

The deflection of a member subjected to concentrated load, *P*, or uniform load, *w*, can be calculated by (1), derived from the Winkler model. Consider

(1) $$\begin{array}{c} EI\frac{d^{4}y}{dx^{4}}+ky=p, \end{array}$$

where *E* is modulus of elasticity, *I* is moment of inertia, *y* is deflection, *k* is modulus of foundation, and *p* is axial force. Since axial force is not applied to beams or slabs, *y* = *e* ^*ax*^ in (1). Therefore, it is found that (1) is satisfied if

(2) $$\begin{array}{c} \left(a^{4}+\frac{k}{EI}\right)y=0, \end{array}$$

where

(3) $$\begin{array}{c} a=\pm \beta \left(1\pm i\right),\\ \beta ={\left(\frac{k}{4EI}\right)}^{1/4}. \end{array}$$

The general solution of (1) can be

(4) y=eβxAcos⁡⁡βx+Bsin⁡(βx)+e−βxCcos⁡⁡βx+Dsin⁡βx,

where *A*, *B*, *C*, and *D* are the constants of integration and can be calculated by load and boundary conditions. In the case of an infinitely long beam supported by a continuous foundation, *x* → *∞* in (4). Therefore, the constants *A* and *B* in (4) must equal zero. Consider

(5) $$\begin{array}{c} y=e^{-\beta x}\left[C\cos\left(\beta x\right)+D\sin\left(\beta x\right)\right]. \end{array}$$

The conditions applicable for a very small distance to the right of the concentrated load, *P*, are

(6) $$\begin{array}{c} -EIy^{\prime \prime \prime }\left(0\right)=-\frac{P}{2},\\ C=D=\frac{P}{8\beta^{3}EI}=\frac{P\beta }{2k}. \end{array}$$

Finally, the introduction of the expressions for the constants in (5) provides the following equations, applicable to an infinite member subjected to a concentrated load or uniform load:

(7) y=∫0lw·dx2k·βe−βxcos⁡(βx)+sin(βx)  (uniform  load),

(8) y=P·β2k·e−βxcos⁡(βx)+sin(βx) (concentrated  load).

**Figure 6:**
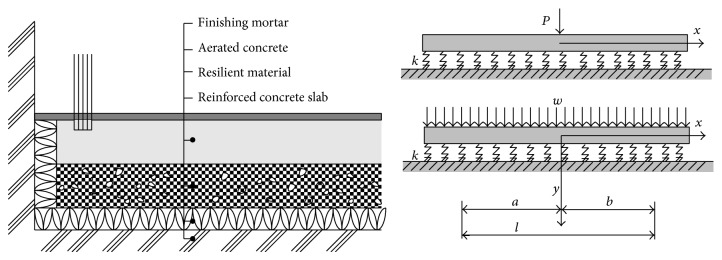
Model of floor sound insulation system.

The floating concrete floors insulation systems were modeled as shown in Figure 6. In the model, reinforced concrete slabs were assumed as a material having infinite stiffness because the modulus of elasticity of the reinforced concrete slab was much greater than that of the other materials such as resilient material, aerated concrete, and finishing mortar. The placing of the resilient material on the slab was modeled as a material supported by springs having constant spring coefficient, *k*. The aerated concrete and finishing mortar are also assumed as a material having infinite stiffness, as shown in Figure 6 [19].

**Figure 7:**
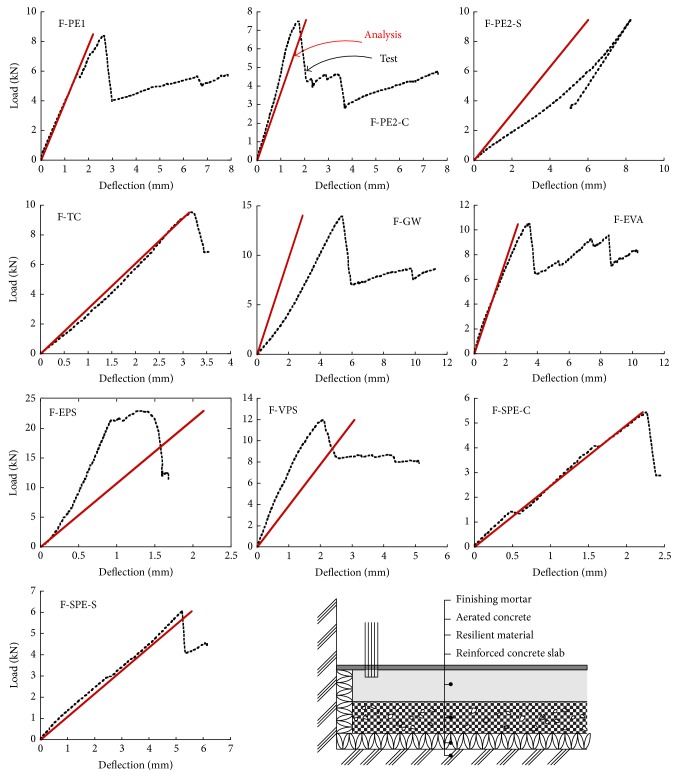
Comparisons of the observed and predicted load versus deflection curves.

**Figure 8:**
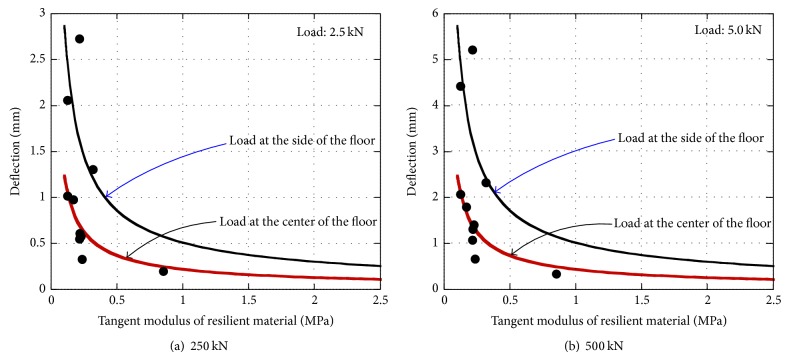
Comparisons of the observed and predicted deflection versus tangent modulus relationships.

The comparison between the experimental and predicted load-deflection behavior of floor sound insulation specimens by the Winkler model is shown in Figure 7. In these figures, only the ascending branch of curves predicted by the Winkler model was included since the model was incapable of predicting the curve after peak load. It was seen in these figures that the Winkler model was able to predict the load-deflection behavior of the floating concrete floors up to the peak load with reasonable agreement. The deflections of test specimens at loads 2.5 kN and 5 kN were also compared with those calculated by the Winkler model as shown in Figure 8. The solid curve represents the deflection of the floating concrete floor subjected to the load at the side or at the center. As shown in the figure, in general, the predicted deflection by the Winkler model was approximately equal to the experimentally observed results. The simulated results indicate that the deflection of the floating concrete floor rapidly increases as the tangent modulus of the resilient material decreases, especially for the floor having a modulus lower than 0.5 MPa. The effect of the location of load on the deflection of the floating concrete floor exhibited trends similar to those of the findings from the experimental studies. When the tangent modulus was 0.5 MPa, the deflection of the specimen subjected to a load of 2.5 kN at the side was about 350% greater than that of the specimen subjected to load at the center, as shown in Figure 8(a).

The cracking strength of the floating concrete floor can also be found by the Winkler model. Differentiation of the deflection of (8) leads to the deflection angle *θ* and curvature *ϕ* of floor as

(9) $$\begin{array}{c} \theta =y^{\text{'}}=-\frac{\beta^{2}\cdot P}{k}\cdot e^{-\beta x}\left[\sin\left(\beta x\right)\right], \end{array}$$

(10) $$\begin{array}{c} \phi =y^{\prime \prime }=-\frac{\beta^{3}\cdot P}{k}\cdot e^{-\beta x}\left[\cos\left(\beta x\right)-\sin\left(\beta x\right)\right]. \end{array}$$

The curvature *ϕ* of floating concrete floor in elasticity can then be calculated from (11) as

(11) $$\begin{array}{c} \phi =\frac{\varepsilon_{\text{cr}}}{h/2}, \end{array}$$

where *ε*_cr_ is the cracking strength of the concrete floor and *h* is the thickness of the floor.

Substitution of the curvatures, given by (11), into (10), leads to

(12) $$\begin{array}{c} P_{\text{cr}}=\frac{\varepsilon_{\text{cr}}}{h/2}\cdot \frac{k}{\beta^{3}e^{-\beta x}\left[\cos\left(\beta x\right)-\sin\left(\beta x\right)\right]}. \end{array}$$

To estimate the cracking strength of the floor, the cracking strain, *ε*_cr_, of concrete proposed by Belarbi and Hsu [20] was adopted, taken as 0.00008. The cracking strength of (12) considering the effect of the modulus of resilient materials predicted the cracking strength of the sound insulation floor, as shown in Table 2, with 1.67 on average and 25.1% in COV.

## 5. Conclusions

In this study, experimental and analytical investigations were conducted to determine the effects of the types of resilient materials and the location of loading on the structural behavior of floating concrete floors. Based on the experimental and analytical results, the following conclusions can be drawn.

1. A total of twenty-seven resilient material tests indicated that the strength of resilient materials was strongly influenced by the types of materials and the bottom shapes of materials. At the beginning of loading, the compressive stress of EPS and EVA specimens increased with little change in slip, while the stresses of the other specimens increased in a slow grade. The stain of the plat-shaped specimens was smaller than that of the embossed-shaped specimens at the same stress stage.
2. Among nine types of tested resilient materials, the tangent modulus of EPS specimen was the greatest, while that of SPE specimen was the lowest. The tangent modulus of all specimens except EPS specimen was approximately constant up to the strain of 0.3.
3. Ten floating concrete floor tests showed that the maximum load significantly increased with increasing the tangent modulus of the resilient material. The deflection of the floor loaded at the side was greater than that of the floor loaded at the center, and this could be attributed to the decreasing of the supporting area against the external load.
4. The analytical results using the Winkler model indicated that the deflection of the floating concrete floor rapidly increased as the tangent modulus of resilient material decreased, especially for the floor having a modulus lower than 0.5 MPa. Therefore, the resilient material of which the tangent modulus is greater than 0.5 MPa is recommended to be used to support the load on the floor.
5. The proposed equation based on the Winkler model predicted the cracking strength of the sound insulation floor with 1.67 on average and 25.1% in COV.

## Figures and Tables

**Table 1:** Specification of resilient material specimens and test results.

Material	Specimens	Thickness (mm)	Bottom shapes	Stress (MPa)			
				Strain 0.1	Strain 0.2	Strain 0.3	Strain 0.4
Polystyrene (PE)	PE-1	20	Plat	0.021	0.038	0.073	0.127
	PE-2	20	Plat	0.020	0.037	0.069	0.121
	PE-3	20	Plat	0.015	0.031	0.058	0.105

Tire chip (TC)	TC-1	30	Embossed	0.044	0.132	0.179	0.225
	TC-2	30	Embossed	0.039	0.124	0.173	0.225
	TC-3	30	Embossed	0.038	0.115	0.166	0.213

Glass wool (GW)	GW-1	20	Plat	0.033	0.064	0.115	0.397
	GW-2	20	Plat	0.026	0.056	0.090	0.161
	GW-3	20	Plat	0.022	0.044	0.076	0.140

Ethylene vinyl acetate (EVA)	EVA-1	20	Embossed	0.022	0.044	0.070	0.211
	EVA-2	20	Embossed	0.014	0.030	0.100	0.137
	EVA-3	20	Embossed	0.010	0.021	0.039	0.108

Ethylene polystyrene (EPS)	EPS-1	20	Plat	0.196	0.233	0.267	0.303
	EPS-2	20	Plat	0.196	0.233	0.263	0.296
	EPS-3	20	Plat	0.182	0.214	0.243	0.269

Vinyl sheet and polystyrene (VPS)	VPS-1	40	Plat	0.036	0.053	0.075	0.111
	VPS-2	40	Plat	0.033	0.050	0.072	0.108
	VPS-3	40	Plat	0.030	0.047	0.069	0.105

Polystyrene (SPE1)	SPE1-1	20	Plat	0.012	0.025	0.041	0.063
	SPE1-2	20	Plat	0.009	0.022	0.039	0.060
	SPE1-3	20	Plat	0.009	0.021	0.037	0.058

Polystyrene (SPE2)	SPE2-1	40	Plat	0.010	0.027	0.043	0.063
	SPE2-2	40	Plat	0.009	0.025	0.041	0.059
	SPE2-3	40	Plat	0.008	0.022	0.038	0.059

Polystyrene (SPE3)	SPE3-1	60	Plat	0.009	0.021	0.037	0.058
	SPE3-2	60	Plat	0.008	0.021	0.036	0.056
	SPE3-3	60	Plat	0.007	0.020	0.034	0.053

**Table 2:** Specification of floating floor specimens and test results.

Specimens	Resilient material				Loading location	Test results		*P*_cr-cal_/*P*_cr-exp_
	Types	Thickness (mm)	Bottom shapes	Tangent modulus (MPa)		*P*_cr-exp_ (kN)	Max. deflection (mm)	
F-PE1	Polystyrene (PE)	20	Plat	0.222	Center	8.50	2.62	1.37
F-PE2-C	Polystyrene (PE)	20	Plat	0.222	Center	7.58	1.73	1.22
F-PE2-S	Polystyrene (PE)	20	Plat	0.222	Side	9.47	8.22	1.44
F-TC	Tire chip (TC)	30	Embossed	0.173	Center	9.53	3.19	1.63
F-GW	Glass wool (GW)	20	Plat	0.322	Center	14.04	5.43	2.06
F-EVA	Ethylene vinyl acetate (EVA1)	20	Embossed	0.232	Center	10.48	3.40	1.67
F-EPS	Ethylene polystyrene (EPS)	20	Plat	0.859	Center	23.00	1.42	1.59
F-VPS	Vinyl sheet and polystyrene (VPS)	40	Plat	0.240	Center	12.00	2.09	2.58
F-SPE1-C	Polystyrene (SPE1)	20	Plat	0.130	Center	5.46	2.27	1.89
F-SPE1-S	Polystyrene (SPE1)	20	Plat	0.130	Side	6.06	5.21	1.18
